# Risque cardiovasculaire et syndrome d’apnées obstructives du sommeil

**DOI:** 10.11604/pamj.2018.29.47.11267

**Published:** 2018-01-18

**Authors:** Abdelmajid Bouzerda

**Affiliations:** 1 Service de Cardiologie, Premier Centre Médico-chirurgical, Université Cadi Ayyad, Faculté de Médecine et de Pharmacie de Marrakech, Maroc

**Keywords:** Syndrome d´apnée du sommeil, risque cardiovasculaire, pression positive continue, Obstructive sleep apnoea, cardiovascular risk, continuous positive airway pressure

## Abstract

Le syndrome d'apnées obstructives du sommeil (SAOS) touche environ 4% des hommes et 2% des femmes d'âge moyen mais beaucoup de ces patients ne sont ni diagnostiqués ni traités. Une morbidité et une mortalité cardio et cérébrovasculaires sont associées aux apnées du sommeil. La notion de lien de causalité s'est confirmée ces dernières années sur des données épidémiologiques, expérimentales et thérapeutiques. La prévalence dans la population générale et l'impact sur la genèse et l'évolution de l'hypertension artérielle systémique et pulmonaire, des troubles du rythme cardiaque, de la maladie coronarienne, de l'insuffisance cardiaque et des accidents vasculaires cérébraux doivent inciter à diagnostiquer et à traiter précocement les troubles respiratoires du sommeil et si possible à les prévenir.

## Abstract (en)

Obstructive sleep apnoea (OSA) affects approximately 4% of men and 2% of women in the middle ages but many of these patients are not diagnosed or treated. Cardiovascular and cerebrovascular morbidity and mortality are associated with sleep apnoea. This causal link has been confirmed in recent years on the basis of epidemiological, experimental and therapeutic data. The prevalence in the general population and the impact on the genesis and evolution of systemic and pulmonary arterial hypertension, of cardiac rhythm disorders, of coronary artery disease, of heart failure and of strokes should encourage clinicians to early diagnose and treat sleep disordered breathing and, if possible, to prevent them.

## Introductionl

Le syndrome d'apnées obstructives du sommeil (SAOS) concerne 2 à 4% de la population générale adulte [1] et comporte des conséquences sur la morbimortalité cardiovasculaire clairement mises en évidence au cours de ces dernières années. Indépendamment de la présence d'une obésité ou d'autres facteurs de risque confondants, le SAS est associé à un risque augmenté d'infarctus du myocarde, d'insuffisance cardiaque, d'hypertension artérielle (HTA), de morbidité cardiovasculaire globale et de surmortalité cardiovasculaire [2]. Il est en outre associé à l'insulino-résistance, indépendamment de l'obésité avec laquelle il semble avoir un effet au moins additif [3], ainsi qu'au syndrome métabolique dont le risque est multiplié par 9 chez l'obèse apnéique [4]. Il n'est pas rare qu'il coexiste, sans être diagnostiqué, chez des patients affectés d'une maladie cardio-vasculaire; il active divers mécanismes pathologiques dont on connaît le rôle nocif sur le cœur et les vaisseaux; il peut être impliqué dans la progression des affections cardio-vasculaires et la résistance aux stratégies thérapeutiques conventionnelles. Ces données doivent motiver tous les acteurs de santé à un dépistage large de ce syndrome et son traitement précoce afin de diminuer la morbimortalité cardiovasculaire qui lui est attribué. Le SAOS pose des problèmes de cout du diagnostic (polysomnographie) et du traitement à long terme (ventilation en pression positive continue).

## Méthodes

L'objectif de notre étude est de Passer en revue, de façon systématique, les interactions du SAOS avec la physiopathologie et les maladies cardio-vasculaires. , en mettant en évidence sa responsabilité dans la survenue d'événements cardiovasculaires majeurs et le rôle pronostique délétère de sa présence. Nous avons consulté la banque de données MEDLINE pour la période allant de janvier 1966 à septembre 2016, à l'aide des mots clés suivants: apnée du sommeil, obésité, hypertension, insuffisance cardiaque, arythmies cardiaques, maladie coronaire, accident vasculaire cérébral, activité sympathique, endothélium, inflammation et pression positive continue (PPC), afin d'identifier les études consacrées au SAOS. Priorité a été donnée aux grandes études prospectives de cohortes et aux essais contrôlés randomisés. Nous avons identifié 51 études et revues originales se rapportant aux anomalies respiratoires liées au sommeil. Les données ont été analysées pour s'assurer de leur pertinence.

## Etat actuel des connaissances

Le syndrome d'apnées du sommeil (SAS) est défini par la survenue au cours du sommeil d'apnées et/ou d'hypopnées. Ces événements respiratoires sont définis par l'interruption (apnée) ou la diminution de plus de 50% (hypopnée) du flux inspiratoire, durant plus de 10 secondes, survenant en nombre supérieur à 5/ heures de sommeil. Les apnées obstructives sont distinguées des apnées centrales selon que les événements respiratoires sont liés à une obstruction des voies aériennes supérieures, ou à un trouble de la commande respiratoire. Selon la définition de l'American academy of sleep medicine (AASM) [5]. La présence des critères cliniques A ou B, et du critère polysomnographique C sont nécessaires au diagnostic: A: somnolence diurne excessive non expliquée par d'autres facteurs; B: deux au moins des critères suivants non expliqués par d'autres facteurs (ronflements sévères et quotidiens, sensations d'étouffement ou de suffocation pendant le sommeil, sommeil non réparateur, fatigue diurne, difficultés de concentration, nycturie (plus d'une miction par nuit)); C: critère polysomnographique ou polygraphique: apnées + hypopnées ≥ 5 par heure de sommeil (index d'apnées hypopnées [IAH] ≥ 5).

**Figure 1:**
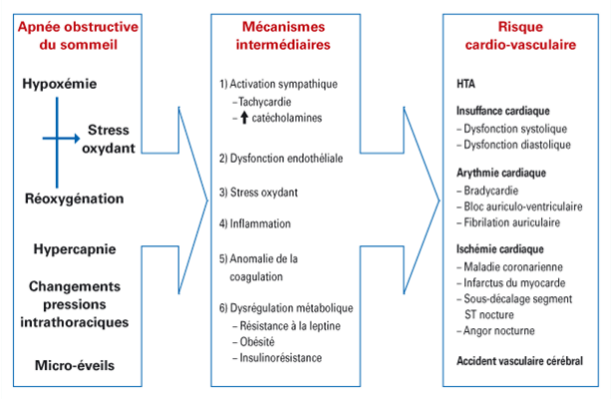
composantes physiopathologiques du syndrome d’apnées obstructif du sommeil impliqués dans le développement des maladies cardio-vasculaires

Le niveau de sévérité sera jugé sur la fréquence de survenue des événements respiratoires anormaux: légère: entre 5 et 15 événements par heure; modérée: entre 15 à 30 événements par heure; sévère: 30 et plus événements par heure (Figure 1).

**Activation sympathique:** Les patients atteints de SAOS ont une hyperstimulation du système nerveux sympathique en direction des vaisseaux sanguins périphériques, y compris durant l'éveil diurne normoxémique [6]. Les raisons de cette hypertonie sympathique demeurent peu claires bien qu'une hyperstimulation des chémo-récepteurs puisse être envisagée [7].

**Dysfonction endothéliale:** L'hypoxémie, l'hypercapnie et les variations tensionnelles qui accompagnent les épisodes d'apnées obstructives sont autant de stimulus puissant de la libération de substances vasoactives, et altèrent le fonctionnement de l'endothélium. Les taux élevés d'endothéline [8] probablement en réponse aux phases d'hypoxémie de l'apnée du sommeil, contribuent à la vasoconstriction soutenue et à diverses atteintes cardiovasculaires .En l'absence de pathologie cardiaque ou vasculaire patente, les patients souffrant de SAOS ont un dysfonctionnement endothélial [9].

**Stress oxydatif et inflammation vasculaire:** L'hypoxie intermittente et les phases de reperfusion concomitantes des épisodes récurrents d'apnée nocturne sont impliquées dans la formation de radicaux libres très réactifs et dans l'apparition de lésions d'ischémie reperfusion au niveau de la paroi vasculaire. Il s'ensuit un risque accru d'athérosclérose [10]. Ce stress oxydant a également pour conséquence une inflammation vasculaire et systémique favorisant le développement de l'HTA et de l'athérosclérose [11]. Cette dernière est accélérée chez les patients SAOS, liée à une interaction entre plusieurs mécanismes physiopathologiques: hémodynamique (HTA), hormono-métabolique (insulinorésistance) et immuno-inflammatoire (stress oxydant, hyperagrégabilité plaquettaire et hypercoagulabilité).

**Coagulation et athérosclérose précoce:** Une hyperagrégabilité plaquettaire est présente au cours du SAOS [12] en partie secondaire à l'élévation nocturne du taux des catécholamines [13]. L'augmentation de l'hématocrite [14], des taux nocturnes et diurnes de fibrinogène [15] et de la viscosité sanguine [16] contribue aussi à la prédisposition à la thrombose et à l'athérosclérose chez ces patients Le SAOS entraîne une athérosclérose précoce, illustrée par une augmentation de l'épaisseur intima-média et la présence de plaque d'athérosclérose au niveau des artères carotidiennes des patients SAOS, mêmes chez ceux indemnes de tout facteur de risque cardiovasculaire [17]. Il a été mis en évidence au cours du SAOS une augmentation du taux d'adipokines pro-inflammatoires plasmatiques qui participent à l'athérosclérose [18].

**Dysfonctions métaboliques:** Les événements respiratoires vont induire une altération du métabolisme lipidique, glucidique et hépatique, indépendamment de l'obésité [19]. L'hyperactivation sympathique induite en est la principale cause comme démontré dans les modèles animaux [20]. L'hypoxie intermittente, en induisant une inflammation du tissu graisseux, entraîne une augmentation des chylomicrons et du LDL-cholestérol circulant favorisant ainsi l'athérogenèse [21]. Également, le SAOS est associé à une fréquence augmentée de l'intolérance au glucose, et du diabète de type 2, indépendamment de l'obésité et de l'âge. Une étude retrouve une prévalence de 50 % et de 30 % respectivement chez les patients SAOS [22]. Le risque cardiovasculaire est donc augmenté chez les patients porteurs d'un SAOS, avec une survenue plus fréquente des événements cardiovasculaires (HTA, insuffisance, arythmie et ischémie cardiaque et AVC). La sévérité de l'hypoxémie nocturne est le facteur majeur prédicteur de ces complications.

**Figure 2:**
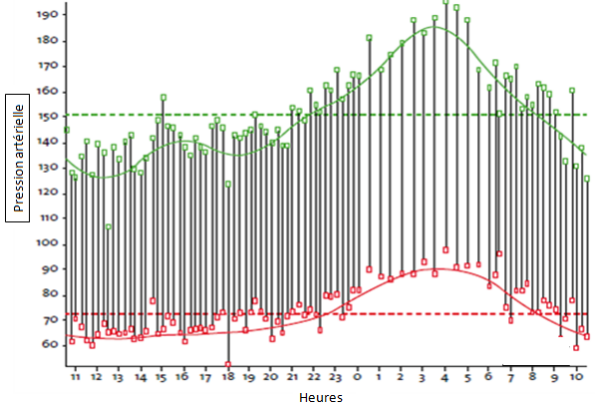
intérêt de la MAPA des 24 heures dans le Syndrome d’apnées obstructif du sommeil

**SAOS et hypertension artérielle systémique:** La prévalence du SAOS est plus élevée dans des populations de patients hypertendus (30-83%) [23]. Le SAOS est un facteur de risque d'HTA indépendamment de l'obésité II a été démontré une relation linéaire entre la pression artérielle des 24 heures et l'index d'apnée-hypopnée, [24] indépendamment de facteurs confondants incluant l'indice de masse corporelle. L'apnée provoque une stimulation sympathique périphérique et centrale (baro et chémorécepteurs). L'asphyxie est évitée par un micro-réveil, qui majore encore l'activation sympathique, et atténue le tonus vagal. L'ensemble se traduit par une augmentation de la fréquence cardiaque et de la pression artérielle, dont les pics peuvent atteindre 240/130mmHg L'HTA associée au SAOS à plusieurs caractéristiques: prévalence importante, prédominance nocturne et diastolique avec fréquemment une diminution nocturne de la pression artérielle systolique ou diastolique inférieure à 10 % (« profil non-dipper »). De ce fait, l'existence d un profil non dipper sur la mesure ambulatoire de la pression artérielle des 24 heures doit faire évoquer la possibilité d'un SAOS (Figure 2). Le SAOS est également fréquent chez les patients ayant une HTA résistante au traitement [25,26]. Il est actuellement reconnu comme une cause d'HTA dans les recommandations américaines [27] et européennes [28] de l'HTA. Sur le plan thérapeutique, L'HTA dans le SAOS est souvent difficile à contrôler pharmacologiquement [29]. Il a été recommandé d'utiliser les bêta-bloquants sur la base d'une évaluation comparative de différentes molécules il y a quelques années [30]. Plusieurs études randomisées ont évalué l'effet du traitement par pression positive continue sur la PA chez des patients avec un SAOS [31-34]. Cet effet est variable selon les études mais semble significatif. Cette réduction de la pression artérielle est plus marquée chez les patients avec un SAOS sévère, des Index de masses corporelles importants, en cas de pressions artérielles élevées à l'état basal et si la compliance au traitement est bonne.

**SAOS et coronaropathies:** l'apnée et l'hypoxémie au cours du SAOS provoquent une inadéquation entre besoins et apports myocardiques en oxygène qui peut provoquer, à la phase aiguë, une ischémie myocardique et déclencher de l'arythmie. A la phase chronique, l'ischémie myocardique est multifactorielle, favorisée par les épisodes récurrents d'hypoxémie, l'hypertension artérielle, la vasoconstriction sympathique, l'augmentation de la pression transmurale, et à plus long terme l'inflammation systémique et la dysfonction endothéliale qui peuvent induire des lésions coronaires. Des études cas témoins ont montré une prévalence élevée de SAOS chez les patients avec une maladie coronaire [35]. De plus, chez les patients avec une maladie coronaire, la présence d'un SAOS est associée à une mortalité plus importante, à plus d'événements cardiaques majeurs et plus de resténose après dilatation coronaire percutanée [36]. Les patients porteurs de SAOS obstructif ont un score calcique plus élevé que les patients sans SAOS, et ce d'autant plus que le SAOS est sévère. Il a également été observé des épisodes nocturnes d'angor et de sous-décalage du segment ST chez près d'un tiers des patients souffrant de ce syndrome. Milleron et al [37] ont montré dans une étude observationnelle que le traitement du SAOS par ventilation en pression positive continue était associé à une diminution de la survenue de nouveaux événements cardiovasculaires.

**SAOS et accidents vasculaires cérébraux:** L'HTA est un facteur de risque reconnu des accidents vasculaires cérébraux (AVC). Outre l'HTA diurne et les variations nocturnes de la pression artérielle systémique, les apnées engendrent des fluctuations de la pression intracrânienne et du débit sanguin cérébral [38]. Parmi les perturbations humorales, l'élévation du fibrinogène plasmatique chez les patients présentant un AVC est corrélée à la gravité du SAOS [39]. Des troubles nocturnes du rythme cardiaque (pauses sinusales, bloc auriculoventriculaire du second degré, tachycardie ventriculaire) sont observés dans près de la moitié des cas d'AVC récents [40]. II a également été rapporté, chez les patients en post-accident vasculaire cérébral, une leucoaraïose plus fréquente en cas de SAOS, suggérant que le SAOS pourrait favoriser ce type de lésions pré-disposantes. Cet effet pourrait passer par l'hypertension artérielle induite par le SAS, dont le profil non-dipper est associé à la leucoaraïose. Les larges études transversales et les études longitudinales conduites sur plusieurs années suggèrent aujourd'hui une relation indépendante entre SAOS et accident vasculaire cérébral, au-delà des nombreuses comorbidités associées, avec une relation dose-effet entre sévérité du SAOS et risque d'accident vasculaire cérébral. De plus, l'existence d'un SAOS en post-accident vasculaire cérébral réduirait le potentiel de récupération, allongerait la durée d'hospitalisation, et augmenterait le risque de récidive et de décès.

**SAOS et arythmies cardiaques:** La prévalence des troubles du rythme et de la conduction est élevée chez les patients ayant Un SAOS, liée à l'élévation de la PA, la dilatation de l'oreillette gauche, l'hypertonie Vagale en réponse aux efforts ventilatoires contre la résistance des voies aériennes supérieures et à l'hypoxémie nocturne, résultant en une modification de la contractilité myocardique. Une étude observationnelle retrouve effectivement une prévalence de 4,8% de fibrillation auriculaire et de 5,3% de tachycardie ventriculaire chez les patients SAS après ajustement [41]. Et inversement, Gami et al. [42] ont retrouvé une prévalence de SAOS de 49% chez les patients en FA contre 32 % dans la population cardiologique témoin. Par ailleurs, le SAOS expose à un risque de récidive de la FA après ablation plus élevé de 25% [43], également, lors des apnées, une bradycardie sinusale sévère est retrouvée chez 5 à 10 % des patients, une pause sinusale ou un bloc auriculo-ventriculaire peuvent également Survenir. Ainsi dans la population de patients porteurs de pacemaker, la prévalence de SAOS non diagnostiquée est de 59%. Sachant que ces troubles du rythme et de conduction régressent habituellement après appareillage du SAOS, la question de savoir si un traitement initial du trouble respiratoire nocturne pourrait éviter la pose du pacemaker n'est pas résolu [44].

**SAOS et insuffisance cardiaque:** Une des conséquences possibles de l'apnée du sommeil est l'insuffisance cardiaque notamment après avoir favorisé la survenue d'événements ischémiques et amené à une altération de la contractilité du myocarde, ou bien par le biais d'un développement de cardiopathie hypertensive voire du stress oxydatif. Quelle que soit l'origine d'une insuffisance cardiaque, en dehors des épisodes de décompensation, cette affection s'accompagne d'une apnée du sommeil pour plus de 50% des patients [45]. Cette apnée peut être obstructive mais aussi et encore plus souvent centrale voire mixte. Pour un insuffisant cardiaque, le fait d'avoir une apnée du sommeil aggrave son pronostic avec une surmorbidité et surmortalité [46]. Ces deux conditions étant majoritairement dues à l'insuffisance cardiaque elle-même, qui s'aggrave donc sous l'influence de l'apnée et peu à des troubles du rythme ou des événements ischémiques cardiaques par exemple. Une autre particularité de l'apnée chez l'insuffisant cardiaque est d'être souvent silencieuse. Les conséquences des SAS notamment sur la morbimortalité cardiovasculaire et accidentelle ainsi que sur la qualité de vie des patients, sont importantes et justifient un diagnostic et un traitement adaptés. Ce dernier est représenté en première intention par la ventilation nocturne, en pression positive avec différentes modalités selon que le SAS est à prédominance obstructive ou centrale. L'observance à cette ventilation passe par une éducation thérapeutique renforcée et une étroite collaboration entre cardiologues et spécialistes du sommeil.

**SAOS et hypertension artérielle pulmonaire:** Une hypertension artérielle pulmonaire diurne existe dans environ 20% des SAOS [47]. Les apnées-hypopnées obstructives s'accompagnent d'élévations transitoires de la pression artérielle pulmonaire, d'autant plus élevées que la baisse de SaO2 est plus importante [48]. Le principal mécanisme évoqué est l'hypoxémie, connue pour induire une hypertension artérielle pulmonaire réflexe par augmentation des résistances vasculaires pulmonaires, et à laquelle les patients porteurs de SAS semblent plus sensibles. Toutefois, le SAOS seul n'induit pas une HTA pulmonaire permanente; celle-ci ne peut se développer que s'il existe une insuffisance respiratoire obstructive ou restrictive associée [49,50]. En pratique, l'HTA pulmonaire ne se rencontre qu'en cas d'association SAS-bronchopathie chronique obstructive ou SAS-syndrome obésité-hypoventilation. Elle peut alors conduire à l'insuffisance ventriculaire droite qui fait partie de la description du syndrome de Picwick.

**SAOS et syndromes aortiques aigus:** Le SAOS favorise la constitution de lésions artérielles, et ce d'autant plus qu'il est sévère. Il existe une relation étroite entre SAOS et syndromes aortiques aigus (SAA), en particulier la dissection aortique [**51**]. Cette relation passe entre autres par la dilatation aortique favorisée par le SAOS. La présence d'épisodes répétés de variation brusque de la pression transmurale, exercée sur la paroi de l'aorte, semble jouer un rôle majeur sur cette dilatation. Tout patient porteur d'un SAOS devrait avoir une recherche de dilatation de son aorte par échodoppler thoracique et abdominal. De même, la recherche d'un SAOS devrait être faite chez tout patient ayant une pathologie aortique [51].

## Conclusion

En raison de l'incidence toujours plus élevée de l'obésité, il faut s'attendre à ce que la prévalence du SAOS continue à augmenter. Les patients ayant une hypertension, des cardiopathies ou des symptômes de troubles de la perfusion vasculaire cérébrale doivent donc être interrogés spécifiquement sur les symptômes du SAOS. La communauté cardiologique doit dépister et traiter le SAOS au même titre que les autres facteurs de risque cardiovasculaire. Nous disposons de traitements efficaces obtenant la disparition des symptômes et probablement l'atténuation du risque vasculaire. La ventilation en pression positive continue est le traitement le mieux évalué aujourd'hui.

### Etat des connaissances actuelles sur le sujet

- Le syndrome des apnées obstructives du sommeil (SAOS) est une maladie sous diagnostiquée qui entraîne une tendance à l'endormissement diurne, des troubles cognitifs et une diminution de la qualité de vie;
- Plusieurs études montrent que le SAOS est un facteur de risque indépendant d'hypertension, d'infarctus du myocarde et d'AVC. Ceci résulte d'une hypoxie intermittente, d'un tonus sympathique augmenté, de troubles des fonctions endothéliales et de différents facteurs neurohumoraux;
- Un traitement du SAOS par pression positive continue non seulement améliore les symptômes mais il permet de corriger une hypertension et peut-être de diminuer le risque de maladies cardiovasculaires et de décès.

### Contribution de notre étude à la connaissance

- Le SAOS est une affection très fréquente dans la population générale et encore plus chez les patients atteints de pathologie cardiovasculaire;
- L'association fréquente entre SAOS et atteintes cardiovasculaires devraient amener la communauté cardiologique à dépister et traiter le SAOS au même titre que les autres facteurs de risque cardiovasculaire;
- L'impact du traitement du SAOS par pression positive continue est actuellement bien démontré.

## Conflits d’intérêts

L'auteur ne déclare aucun conflit d'intérêts.
